# Revealing the Reversal Effect of Galangal (*Alpinia galanga *L.) Extract Against Oxidative Stress in Metastatic Breast Cancer Cells and Normal Fibroblast Cells Intended as a Co- Chemotherapeutic and Anti-Ageing Agent

**DOI:** 10.31557/APJCP.2020.21.1.107

**Published:** 2020

**Authors:** Faradiba Nur Faradiba Nur, Nadzifa Nugraheni, Irfani Aura Salsabila, Sari Haryanti, Muhammad Da’i, Edy Meiyanto

**Affiliations:** 1 *Cancer Chemoprevention Research Center, *; 2 *Department of Pharmaceutical Chemistry, Faculty of Pharmacy, Universitas Gadjah Mada, *; 3 *Medicinal Plant and Traditional Medicine Research and Development Centre, Ministry of Health, *; 4 *Faculty of Pharmacy, Universitas Muhammadiyah Surakarta, Indonesia. *

**Keywords:** Alpinia galangal, co-chemotherapeutic agent, oxidative stress, anti-ageing

## Abstract

**Objective:**

This study intends to explore the potential of galangal extract as a co-chemotherapeutic agent through the analysis of its cytotoxic and migratory effects on metastatic breast cancer cells and as an anti-ageing agent through its senescence inhibitory effect on normal fibroblast cells.

**Methods:**

Galangal ethanolic extract (GE) was subjected to a cytotoxicity test with the 3-(4,5-Dimethylthiazol-2-yl)-2,5-diphenyltetrazolium bromide (MTT) assay alone or in combination with doxorubicin (Dox) against 4T1 cells but not in NIH-3T3 cells. Evidence of senescent cells was detected using a SA-β galactosidase based assay. In addition, the level of reactive oxygen species (ROS), apoptosis, and cell cycle were measured with a flow cytometry-based assay. Meanwhile, cell migration and matrix metalloproteinase (MMP)-9 expression after GE treatment on 4T1 cells were measured using the scratch wound healing assay and gelatin zymography assay, respectively. The metabolomic profiles of GE were traced using gas chromatography-mass spectrometry (GC-MS) analysis.

**Results:**

GE effectively inhibited the growth of 4T1 cells with an IC_50_ value of 135 µg/mL and increased the cytotoxic effect of Dox at concentrations of 50 and 100 µg/mL. GE increased the number of senescent cells arrested in the G2/M phase but did not cause apoptosis. This effect is compounded by increasing intracellular levels of ROS. However, GE reduced senescence to normal in fibroblast cells (NIH 3T3 cells) under oxidative stress by Dox without any changes in the ROS level. Moreover, GE also inhibited the migration of 4T1 cells and suppressed the expression of MMP-9 induced by Dox.

**Conclusion:**

Galangal has the potential for use as a co-chemotherapeutic agent by inducing senescence in correlation with increasing intracellular ROS toward metastatic breast cancer. However, the effect of GE in decreasing the senescence phenomena toward normal fibroblast cells illustrates its potential as a promising anti-ageing agent.

## Introduction

Galangal (*Alpinia galanga* L.) is known as a spice plant in Asian countries. In addition, this plant is commonly used and is mixed as herbal medicine for several diseases (Lo et al., 2013). The potential of this herb for medical use has been explored extensively, including the findings of its active ingredients. Some pharmacological potential can be noted regarding its use as an antimicrobial, anti-inflammatory, antifungal, antioxidant, and anti-osteoarthritic agent (Chouni et al., 2018). Galangal’s pharmacological effects correlate with its active ingredients, including phenylpropanoids, terpenoids, and flavonoids. This herb has also been reported to show anticancer activity, such as inhibiting tumour growth in vitro (Meiyanto and Larasati, 2019). Because some active components show different physiological effects on cancer cells (Chouni et al., 2018), each of the ingredients likely has a different target. Moreover, although the active ingredients of galangal possess antioxidant properties, they increase intracellular ROS that seems to interfere with molecular tumorigenesis. Therefore, it is essential to know the underlying physiological mechanisms of this herb to understand the health benefits of maintaining healthy tissues and inhibiting cancer cells.

The physiological process of cell development between cancer cells and normal cells is the same, namely through the cell cycle (Kosaka et al., 2012). However, cancer cells spend more time in cell division compared with normal cells. Cancer cells also show physiological changes in the process of migration and cell differentiation (Hanahan and Weinberg, 2011). As a result, cancer cells carry out different platforms in energetic metabolism for their survival. This unique platform of cancer metabolism is an interesting focus on the development of anticancer and anti-ageing, especially focusing on ROS metabolism (Meiyanto and Larasati, 2019; Davalli et al., 2016). Some chemotherapeutic agents target ROS metabolisms, such as doxorubicin (Dox) and cisplatin (Yang et al., 2018). Curcumin, a natural compound from Curcuma sp., also targets ROS metabolism. Curcumin increases cellular ROS level by inhibiting ROS metabolic enzymes, which results in the inhibition of the growth of cancer cells but not of normal cells (Larasati et al., 2018). This phenomenon enables us to understand that high levels of ROS in cancer cells are a critical point to target anticancer development without having a significant effect on normal cells.

Galangal, in the form of ethanolic extract or isolated compounds, shows a cytotoxic effect on several cancer cells. Namely ACA (1-Acetoxychavicol Acetate) and galangin, the two major compounds of galangal, exhibited cytotoxic properties in breast cancer cells (Liu et al., 2018), hepatocarcinoma cells (Zhang et al., 2012; Kato et al., 2014), leukemic cells (Bestwick and Milne, 2006), and gastric cancer cells (Hadjzadeh et al., 2014). Apoptosis is the common pathway for its cytotoxicity against cancer cells (Baig et al., 2016). In addition, galangin demonstrated an anti-metastatic property by inhibiting cell migration and MMP expression (Chien et al., 2015). However, it is still unclear whether those phenomena correlate with the increase in intracellular ROS and cell cycle reprogramming.

Cancer cells comprise several hallmarks that might be selectively targeted by anticancer agents. Inverted expression of matrix metalloproteinases (MMPs) and tissue inhibitors of metalloproteinases (TIMPs) serve as key regulators in cancer progression, invasion, and metastasis (Park et al., 2015) Cytotoxic effects are thought to occur through two possible pathways, namely apoptosis and senescence. In response to oxidative stress that could originate from cytotoxic agents, cells commonly undergo cellular senescence, a particularly stable type of cell cycle arrest. Cell cycle arrest also correlates with apoptosis in several respects, presumably mitotic disorders that lead to apoptosis pass through a gradual process of physiological and morphological changes, including senescence. Moreover, apoptosis is regulated by genes that are involved in cell cycle progression (Hanahan and Weinberg, 2011). 

Meanwhile, cell cycle development, apoptosis, cell migration, and the phenomenon of cell ageing show a correlation with increased levels of intracellular ROS that work through specific signaling pathways (Sznarkowska et al., 2017). Cancer cells are more sensitive to ROS accumulation than normal cells are so that a significant elevation in ROS levels will disrupt only cancer cells but not normal cells (Zhong et al., 2019). Interestingly, GE possesses both cytotoxicity and radical scavenging abilities that reduce intracellular ROS. These biological effects seem to contradict each other, but maybe useful for disrupting cancer growth and maintaining cellular stability in healthy tissues. Hence, these different effects depend on the particular characteristics of the cells that distinguish between cancer cells and normal cells. All the studies of GE exert a potent, selective effect on physiological events that occur in these different cells. Therefore, we investigated the effect of GE by revealing its selectivity for cancer cells compared with normal cells and focused on the phenomena of changes in oxidative stress-based physiology. This study could be developed to obtain the potential pharmacological benefits conferred by galangal when used for cancer co-chemotherapy and anti-ageing. 

## Materials and Methods

*Galangal Extract (GE) Preparation*

Galangal (*Alpinia galanga *L.) was obtained from Medicinal Plant and Traditional Medicine Research and Development Centre, Tawangmangu, Ministry of Health, Republic of Indonesia. It was mashed, extracted with 96% ethanol, and the dried GE was collected for the experiment.

*Cell Culture*

4T1 breast cancer cells (ATCC® CRL-2359) and NIH-3T3 (ATCC^®^ CRL-1658) fibroblast cells were obtained from Professor Masashi Kawaichi, Nara Institute of Science and Technology, Japan. The cells were cultured in Dulbecco’s modified Eagle’s Medium supplemented with fetal bovine serum 10% and 1% penicillin-streptomycin under standard conditions (37^°^C, 5% CO_2_). 

*MTT Assay*

Cells were seeded into each well of a 96-well plate at a concentration of 3 x 10^4^ cells/mL and treated with various concentrations of GE in combination with DoxS (Sigma) for 24 h. After incubation, the media were discarded, and cells were washed 1X with phosphate-buffered saline (PBS). Then, MTT reagent (Sigma) was added and incubated for 4 h. SDS was added, incubated overnight, and then the absorbance was measured on a microplate reader (Bio-Rad) with λ = 595 nm.

*Cell Cycle Analysis*

Cells (2 x 10^5^ cells/well) were distributed onto a 6-well plate and incubated for 48 h. Cells were treated with various concentrations of GE, Dox, and a combination of both agents, and incubated for 24 h. Floating and attached cells were collected and pelleted at 2,000 rpm for 3 min, followed by washing with PBS. The cell pellets were suspended in 50 µg/mL propidium iodide in the presence of 100 µg/mL RNase and triton x solution and incubated for 10 min at 37^o^C in the dark. Cell cycle distribution was analyzed by flow cytometry using a BD Accuri C-6 flow cytometer. The percentage of the cell population in different phases of the cell cycle was determined by BD Accuri C6 software.

*Annexin V-PI Staining Apoptosis-based Assay*

Cells were treated with different concentrations of GE, Dox, and a combination of both compounds for 24 h. Then, they were harvested, washed with PBS, and resuspended in PBS. Cells were stained with Annexin V-FITC/PI according to the protocol of Annexin V-FITC cell Apoptosis Detection Kit (Roche). After incubation, the samples were measured with a BD Accuri C-6 flow cytometer and analyzed using BD Accuri C-6 software. 

*SA-βGal Senescence-based Assay*

Expression of β-galactosidase related to senescence was traced through the SA-βGal assay. Cells (1.5 × 10^5^ cells/well) were seeded into each well of a 6-well plate and incubated for 24 h. Cells were washed with 1X PBS twice. Then a fixation buffer was added, allowed standing time, and then washed using 1X PBS once. An additional 1–2 ml of X-Gal solution was added and then incubated at 37ºC. Cells were observed after 72 h under a microscope (CKX-41 Olympus) with a magnification of 200x. When blue cells appear, they are galactosidase positive cells as senescent cell representations (Debacq-Chainiaux et al., 2009).

*DCFDA Staining ROS-based Assay*

Changes in the intracellular level of reactive oxygen species were monitored using the fluorescent probe DCFDA, which is a specific probe for hydrogen peroxide. Cells were distributed in a 24-well plate and incubated for 24 h. After that, 4T1 and 3T3 cells were treated with GE, Dox, and a combination of both agents, before treatment with 10 mM of DCFH-DA at 37°C for 30 min. Cells were collected, washed, and resuspended in supplemented buffer. The DCF fluorescence of at least 20,000 cells was detected and analyzed after 4 h incubation using flow cytometry (BD Accuri C6 flow cytometer) with an excitation wavelength set at 488 nm and an emission wavelength set at 525 nm.

*Scratch Wound Healing Cell Migration-based Assay*

Cell migration was traced through the scratch wound-healing assay. Cells (8 × 10^4^) were seeded into each well of 24-well plates, incubated for 24 h and were starved with serum-free medium for 18 h. Then, cells were scratched with a yellow pipette tip and treated with GE, Dox, and a combination of both agents. Scratched cells were documented at 0, 24, and 48 h. Quantification of the area of wound closures was calculated using ImageJ software.

*Gelatin Zymography MMP-9 Expression-based Assay*

MMP-9 expression was traced through the gelatin zymography-based assay. Cells (2 x 10^5^) were seeded into each well of 6-well plate then incubated for 24 h. The cells then were given GE, Dox, or a combination of both agents in serum-free medium. The media were collected and subjected for gel electrophoresis (PAGE) on 10% SDS-PAGE gel containing 0.1% gelatin under conditions of 100 volts and 50 A for 2 h. The gel was denatured with a denaturing solution containing 2.5% Triton X-100 for 30 min and incubated with incubation buffer for 20 h, and then stained with 0.5% Coomassie Brilliant Blue and incubated for 30 min. After 30 min, the gel was destained by a destaining solution. The gel was scanned and documented. Band intensities were measured using ImageJ software.

*GC-MS Analysis Metabolomic-based Assay*

Metabolomic profiles of GE were analyzed using GC-MS (Shimadzu single quadrupole GC-MS-QP2010 Ultra gas chromatograph-mass spectrometer) using a 30 m x 0.25 mm RP-5 non-polar column (Shimadzu) with 0.25 μm film thicknesses. The helium mobile phase was piped through the column as a carrier gas; the flow through the column was at the rate of 0.55 mL/min, with the split ratio getting up to 139. A sample volume of 0.2 ul was injected into a column set at 40°C for 10 minutes, increased to 180°C at a rate of 2.5°C/minute, then remained at this speed for 20 minutes. While MS was running with an electron impact ionization mode with ionizing energy of 70 eV, it scanned from m/z 28.00 to 600.00 at 0.5 scans/sec. The ion source temperature was 150°C, and the quadrupole temperature was 300°C, while the electron multiplier voltage was maintained at 1.5 kV. The retention time and retention indices for each compound were confirmed by Wiley MS Libraries. 

*Statistical Analysis*

Data presented as mean ± SD, followed by statistical analysis using Student’s t-test. P-values (*p < 0.05; **p < .01) are included in each experiment Figure. 

## Results

*Cytotoxic Effect of GE on 4T1 and NIH-3T3 Cells*

**Figure 1:**
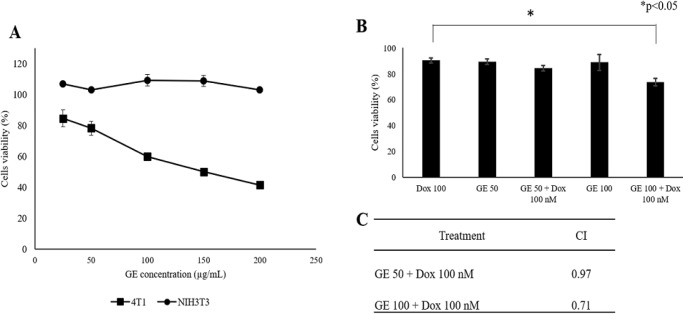
Cytotoxic Effect of GE on 4T1 and NIH-3T3 Cells. Cytotoxic effect was evaluated based on cell viability after treatment with GE alone and in combination with Dox. 4T1 cells (3 × 10^3^ cells/mL) and NIH-3T3 (1 × 10^4^ cells/mL) were treated with GE 1–200 µg/mL for 24 h then subjected to be evaluated by MTT assay. (A) The effect of GE treatment on growth profiles of 4T1. (B) The effect of GE treatment on growth profiles of NIH-3T3. (C) The cell viability profiles of 4T1 cells treated with GE alone and in combination with Dox (n = 3). (D) The combination index (CI) values of GE and Dox treatment. Significant differences between the two treatments were determined using a t-test

The aim of this study is to examine the effect of GE on several hallmarks of cancer and their selectivity for normal cells in vitro. First, we examined the cytotoxic effect of GE toward 4T1 and NIH-3T3 cells. We used 4T1 cells as a model of highly metastatic triple-negative breast cancer (Pulaski Beth A. and Ostrand-Rosenberg Suzanne, 2001) and NIH-3T3 as a model for normal fibroblast cells. The GE with the concentrations of 20–200 µg/mL decreased cell viability against 4T1 cells (Figure 1a) in a dose-dependent manner with IC_50_ values of 135 µg /mL. Therefore, GE is considered a potential weak cytotoxic agent (Prayong et al., 2008). While up to a concentration of 200 µg /mL, GE did not show a cytotoxic effect on NIH-3T3 fibroblast cells. This result demonstrated that GE has a selective cytotoxic effect toward cancer cells with a selectivity index >2.

*Effect of GE on Cell Cycle Profile and Apoptosis*

**Figure 2:**
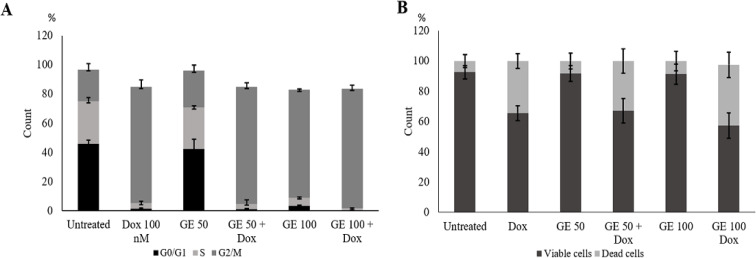
Cell Cycle Distribution and Apoptotic Effect of GE Treatment, Alone and in Combination with Doxorubicin. 4T1 cells (2 × 10^5^ cells/mL) were treated with GE 50 and 100 µg/mL and in combination with Dox 100 nM for 24 h and subjected to cell cycle analysis with PI staining with flow cytometry or Annexin V/PI staining with flow cytometry (n = 3). (A) Cell cycle distribution profile. (B) Apoptotic evidence profile. Significant differences were determined using a t-test

Hence, we inferred that GE is cytotoxic toward breast cancer and possibly interferes with the cell growth program like the ACA (GE‘s main compound) effect. This effect may induce cell cycle arrest in MCF-7 cells in the G1 phase (Awang et al., 2010). We then examined the effect of GE on cell cycle progression using flow cytometry. We found that GE alone induced G2/M cell accumulation, and the G2/M cell population increased when GE 100 µg /mL was given in combination with Dox (Figure 2a). As the characteristic feature, cell death differs based on its process, namely apoptosis, autophagy, and necrosis.

Furthermore, the phenomenon of decreased cell viability was confirmed by observing the induction of apoptosis to determine whether cell death might occur through the apoptotic pathway. However, using annexin V/PI staining-based flow cytometry, GE 50 µg/mL and 100 µg/mL alone and in combination with Dox did not significantly increase cell death (Figure 2b). These phenomena inform us that the increasing cytotoxic effect of GE to Dox at an early time tends to enhance cell cycle arrest rather than apoptosis. During cell arrest, cells might prepare by cell physiological changes, for instance, senescence. Thereby, it could be deduced that a part of decreasing cell viability at a concentration of 100 µg/mL may be due to other related pathways that need to be explored.

*Senescence Induction Following GE Treatment on 4T1 Cells*

To comprehend our understanding that cell cycle arrest closely correlates with cellular senescence, we then investigated the effect of GE on cellular senescence using a β-galactosidase based assay. In this experiment, we used Dox as a positive control as well as a known senescence-inducing agent (Joiner et al., 2006). Figure 5 indicates that the untreated cells induced senescence in as much as 5%, Dox 20%, GE 50 ug/ml 15%, GE 100 ug/mL 25%, and the combination of GE 50 and 100 ug/ml Dox 30% and 45%, respectively. These results suggested that GE 50 and 100 µg/mL significantly increased (p < .01) senescent cells compared with untreated cells, while the combination of GE and Dox also increased significantly (p < .01) compared with Dox treatment. Therefore, these results indicated that cell cycle arrest correlates with senescence. In addition, cell senescence might occur in both normal and cancer cells due to natural selectivity effects. 

*Confirmation of Senescence: Evidence in NIH-3T3 Cells with GE Treatment*

This study showed that GE affects G2/M arrest and the senescence phenomena in 4T1 cells. However, the use of natural products as anticancer agents is often not selective only for cancerous cells, and they might affect normal cells. To ensure the selectivity of GE effects toward cancerous cells, we investigated the effect of GE toward the senescence phenomenon on normal cells using NIH-3T3 cells. The NIH-3T3 is a fibroblast cell that constructs a cellular matrix to support skin flexibility. The results showed that GE treatment did not show any changes in β-galactosidase-positive cells compared with untreated cells. This result suggested that GE at 50 and 100 µg/mL exhibited safe conditions for the cells. 

In contrast, Dox significantly induced several β-galactosidase-positive cells indicating that Dox increases the number of cells in senescence (p < 0.01), which may correlate with its property of generating oxidative stress in normal cells. However, interestingly, the treatment of GE to the Dox-treated cells showed a significant reduction in the number of β-galactosidase-positive cells (p <0 .01) (Figure 4). These results indicated that GE is selective for cancer cells regarding the induction of senescence without any damage to normal cells. However, cellular senescence is a complex process, in which oxidative stress commonly occurs. In this case, the increase of intracellular ROS is believed to be the leading cause of senescence (Larasati el at, 2018). In this regard, the changes in ROS expression with treatments need to be investigated further.

*Effect of GE on ROS Level in 4T1 and NIH-3T3 Cells*

ROS is a byproduct of metabolism that usually correlates with the principle of oxidative stress, induces pathological conditions in an organism through the destruction of lipids, proteins, and DNA. A high level of ROS is a common hallmark found in most cancer cells, where ROS plays a role in supporting various aspects of tumour growth and development. However, the level of ROS that exceeds the threshold in cancer cells leads to the phenomenon of apoptosis and senescence through activation of the p53 pathway (Hanahan and Weinberg, 2011). 

Since GE at 50 and 100 μg/mL both alone and in combination with Dox causes an increase in senescent cells in 4T1 cells, we investigated the potential involvement of GE in the ROS pathway, which might implicate the phenomenon of senescence. In this study, we used Dox as an inducer of intracellular ROS production. In DLD-1 human colorectal cancer cells, Dox caused the oxidant-antioxidant imbalance through induction of oxidative stress (Yokoyama et al., 2017). In cancer cells, Dox promotes excessive production of intracellular ROS (Wang and Yi, 2008). Therefore, in this study, 4T1 and NIH-3T3 cells were treated with GE at a concentration of 50 and 100 μg/mL alone and in combination with Dox 100 nM. Figure 5a shows that Dox and the combination of GE and Dox treatments increased the ROS level significantly. Meanwhile, in NIH-3T3 cells, GE 50 and 100 μg/mL alone and in combination with Dox 100 nM did not interfere with the ROS level (Figure 5b). This result indicated that there was an inter-relationship among ROS, senescence, and cell cycle arrest that mainly existed in cancer cells. 

*Combination of GE and Doxorubicin in Migration Effect in 4T1 Cells*

Our data indicated that GE induced senescence and elevated ROS levels. Since oxidative stress is also as an initiator of cell migration that implicates the metastatic phenomenon (Hanahan and Weinberg, 2011); we then observed whether GE interferes with this process. It is well known that Dox could trigger cell migration through the formation of lamellipodia at a concentration of 10 nM (Amalina et al., 2017). Therefore, we used this compound as a positive control or the co-chemotherapeutic challenge of GE. 

Metastasis consists of complicated steps, which can be inhibited molecularly by a compound. Cell migration, one of the stages of metastasis, is the critical step in the formation of secondary tumours in other organs (Polacheck et al., 2013) thereby making it important to be explored first as an anticancer agent targeting metastasis. Using migration in a wound closure-based assay, we found that GE 25 µg/mL in combination with Dox 10 nM inhibited cell migration significantly (p < 0.05) (Figure 6b) irrespective of cell viability at the IC_50_ values of this combination. In this experiment, we used GE at a low dose (25 µg/mL) to ensure that at this dose, GE is not cytotoxic to cells (Figure 6a). The result showed that cell morphology after 24 and 48 h scratching (Figure 6a) both in cell control and Dox formed lamellipodia, but not in the GE treatment. The data showed that GE exhibited a migratory inhibitory effect on cancer cells. 

*Effect of GE and Doxorubicin on MMP-9 Expression in 4T1 cells*

Based on the cell migration assay, GE is able to inhibit cancer cell migration. However, it needs to be examined further through protein expression involved in the process of metastasis. MMP-9 is a gelatinase that is highly expressed by cancer cells to degrade the ECM and facilitate their migration (Huang, 2018). MMP-9 has become our focus. Before the MMP-9 expression effect assay, we performed a cytotoxic combination test of GE and Dox to 4T1 cells. The combination of Dox and GE reduced cell viability by 80% (Figure 7a.). However, there were no differences between control cells and Dox treatment. The band formed is the result of MMP-9 activity in degrading the gelatin as the substrate (Figure 7b). In accordance with the migratory inhibitory effect, GE suppressed MMP-9 expression that involved the metastatic process (Mehner et al., 2014).

*Metabolomic Analysis of GE*

Since the pharmacological effect of a natural product is close to its metabolite content, we analyzed the metabolomic of GE using GC-MS for volatile oils. A total of 39 components were identified in GE, representing about 100% of the total volatile oils. The major components of the essential oils were beta-sesquiphellandrene (17.25%), beta-bisabolene (13.95%), and beta-caryophyllene (7.27%) (Figure 8 and Table 1). Wu et al., (2014) and Singh et al., (2004) studied the essential oil components of some galangal species and found that the major components differed from the major components possibly because of differences in cultivating conditions. Due to the different sources of galangal, galangal may exhibit differences in cytotoxic activity (Suhendi et al., 2017). We found that the primary compounds in this extract were known to have anticancer activity and antioxidant properties (Table 1). Likewise, we learned that at least a phenylprophane compound is also known to possess antioxidant properties and cytotoxic activity against some breast cancer cells (Dai et al., 2019). As a result, the GE effect on cancer and normal cells in concern with the metabolic profiles needed to be explored further.

## Discussion

Although galangal has been explored for many pharmacological benefits, especially its anti-cancer properties, it is important to know the critical point where galangal distinguishes between its effects in maintaining healthy cells and eliminating those that are aberrant. With this as our focus, we evaluated the evidence-based cytotoxic properties of the physiological changes caused by galangal on metastatic breast cancer cells, which were shown by 4T1 cells as opposed to normal fibroblast cells, and were shown by NIH-3T3 cells. The results showed that galangal, in the form of ethanolic extract (GE), had a cytotoxic effect on 4T1 cells, but was less cytotoxic to NIH-3T3 cells with a selectivity index >4. This finding indicated that GE was more selective for cancer cells than normal cells. In addition, this result provides a general description that galangal is indeed safe to be used as a part of a daily lifestyle to taste the food. The content of compounds that have antioxidant properties can add value to their use to maintain a healthy body. Galangal ethanolic extract used in this study represents most of the content of galangal active compounds, including the volatile oil content. GC-MS analysis indicated the presence of compounds that are common in galangal, for example, eugenol and its derivatives, sesquiterpenes, and coumarins (Table 1), are widely reported to have cytotoxic and antioxidant properties. The content of these compounds supports the potential effects of this GE as an anticancer agent, and at the same time, protects cells from oxidative damage.

In a more in-depth study of changes in cell physiology due to the administration of extracts showed impressive results toward the selectivity of cancer cells. GE stimulates cell cycle arrest, which seems to be associated with the induction of senescence because of an increase in ROS level in cancer cells. This event is not found in normal cells, so it is likely that it is the basis of the differences in cytotoxic responses between cancer cells and normal cells. Cellular senescence is a physiological process of irreversible cell cycle arrest that contributes to various physiological and pathological processes of ageing but can be evaded in cancer cells. This is a new alternative and therapeutic strategy for cytotoxic treatment. It leads to focusing on the target of a cytostatic approach to cancer therapy and ageing or ageing-related diseases (Naylor et al., 2013). Although senescent cells have irreversibly lost their capacity for cell division, they are still viable, remain metabolically active, and subsequently undergo apoptosis (Marin et al., 2017). Hence, developing a pro-senescence agent in relation to the ROS generation for cancer therapy can be a cancer prevention therapeutic strategy. 

Metastatic cancer cells are known to perform the Warburg effect phenomenon, in which the cells metabolize glucose under lack of oxygen resulting in partial glycolysis to produce lactic acid (Han et al., 2013). In this process, cancer cells produce much more ROS in cells, causing oxidative stress conditions. However, cancer cells express a higher level of ROS metabolizing enzymes to neutralize the hazards of ROS, allowing the cells to survive and grow, and avoiding cell cycle arrest (Sullivan and Chandel, 2014). This platform event is also commonly found in cancer stem cells (Zhong et al., 2019). This phenomenon differs when compared with normal cells that undergo normal oxidative metabolism with a very low intracellular ROS level (Ray et al., 2012). Increasing the ROS level in cancer cells is dangerous because it can damage cells (Schieber and Chandel, 2014). Our data support this and show that GE treatment increases the ROS level and senescence in 4T1 cells but not in NIH-3T3 cells. More interestingly, GE enhances ROS expression in cancer cells in response to Dox treatment. 

In contrast, GE did not affect the ROS level in normal cells in single and slightly decreased ROS level against Dox treatment. These occurrences suggest that GE may work to increase the ROS level effectively in cancer cells by interfering with ROS metabolizing enzymes such as GST and CYP (Bezerra et al., 2017), whereas such enzymes have no significant roles in normal cells. An increase in ROS in the cells due to the administration of GE is the key to the important mechanism of its cytotoxic properties in cancer cells. Some previous studies revealed that the phenylpropanoid compounds in GE can inhibit cell proliferation in lung cancer (Lakhsmi et al., 2019), hepatocarcinoma (Kato et al., 2014), and breast cancer (Samarghandian et al., 2014) by inducing cellular growth arrest through the induction of ROS-dependent premature senescence (Khan et al., 2012). Since there are so many types of antioxidant enzymes that play roles in ROS scavenging in the cells, further exploration of the presence of compounds in GE that can interact with particular antioxidant enzymes in cells is needed.

The occurrence of both elevated ROS levels and senescence following combination treatment with GE and Dox also prompted us to consider that GE may have the potential to be developed as a co-chemotherapeutic agent. Co-chemotherapeutic agents are ingredients, both of which originate from nature or from synthetic compounds, which can increase the cytotoxic effect and reduce the side effects of chemotherapeutic agents (Meiyanto et al., 2012). Doxorubicin used in this study is a chemotherapeutic agent known to increase ROS and senescence of cancer cells (Joiner et al., 2006; Wang and Yi, 2008). However, this increase in ROS and senescence does not only occur in cancer cells but may also happen in normal cells. The increase in ROS and senescence in cancer cells is meaningful to its cytotoxic effects, while this same increase in normal cells results in damage to normal tissue causing adverse side effects. GE is a representation of a natural product, which in this study, showed a reversal effect in combination with Dox against cancer cells and normal cells. The effects shown reinforce the basis of the use of GE as a co-chemotherapeutic agent with Dox. Namely, GE can increase the impact of Dox’s cytotoxic effects on cancer cells and at concurrently can reduce its side effects in normal cells. Therefore, further research focusing on underlying molecular mechanisms is needed to comprehend the evidence-based therapeutic uses of galangal.

Metastatic cancer cells have characteristic similar to cancer stem cells. In conditions of stress due to lack of oxygen, metastatic cancer cells survived through anaerobic metabolism that produces radicals. Therefore, metastatic cancer cells tend to produce excessive ROS. Cancer treatment, in general with chemotherapy, only triggers a stress condition in cancer cells. In typical cases, metastatic and cancer stem cells can survive against stress conditions and exhibit resistance to chemotherapeutic agents, so the cancer cells relapse with more virulence after chemotherapy (Vinogradov and Wei, 2012). Therefore, the development of co-chemotherapeutic agents that can overcome the phenomenon of cancer cell relapse that is resistant to chemotherapeutic agents is an important issue. Metastasis consists of multiple stages that begin from invasion and cell migration characterized by the formation of lamellipodia to support cell motility (Machesky, 2008). Our study revealed that the treatment of a single GE in combination with Dox inhibited 4T1 cell migration. This effect is interesting because GE could inhibit Dox stress-induced metastatic cancer cells. Therefore, this indicates that GE provides an alternative way to overcome the metastatic process of cancer cells induced by oxidative stress. Furthermore, GE alone and in combination with Dox suppressed the expression of MMP-9, the marker protein involved in metastasis. Thus, GE may be developed as a co-chemotherapeutic agent targeted at inhibiting metastasis. 

Additional findings of the senescence effect indicated that GE was able to lower the senescence cell number in normal fibroblast cells even though they were induced by stressors such as Dox. Human fibroblast cell ageing is a progressive process that includes intrinsic ageing and extrinsic-photo damage, both of which cause an accumulation of reactive oxygen species (ROS), resulting in dermal fibrosis dysfunction and wrinkle formation (Wen et al., 2017). Increased levels of intracellular ROS that exceed the limit disrupt cell homeostasis, causing DNA damage, so it limits cell replication (Chandrasekaran et al., 2017). Thus, the effect of GE in lowering senescence has the potential to maintain the continuity of normal cell replication to improve body fitness. Some previous findings support these results. Namely, both ACA and galangin found in *Alpinia* sp. protect human dermal fibroblasts from senescence by inhibiting NF-κB activation, decreasing the expression of inflammatory factors, and upregulating IGF1R/Akt-related proteins. These actions indicate that galangin may be a potential candidate for developing natural anti-ageing products that protect the skin from damage caused by ROS (Wen et al., 2017). The ethanolic extract of galangal has a relatively stable antioxidant activity (Chouni and Paul, 2018). Most chemical components of GE (Table 1) have cytotoxic activities that possibly have a synergistic effect of inhibiting cancer cell proliferation. Even though the reversal effect is a common feature in natural products (Meiyanto et al., 2018), the active compounds from GE that play a role in reducing senescence and are needed by normal cells have to be explored further. Thus, additional research is required to determine the active compounds of GE that play specific roles in anti-senescence activity to gain optimal benefits. 

Taken together, in the present study, we found that GE proved to be selectively cytotoxic toward 4T1 cells and enhanced the cytotoxic effect of a chemotherapeutic agent. In addition, GE exerts potential anti-metastasis activity by suppressing MMP-9 expression and inhibiting the migration of highly metastatic breast cancer 4T1 cells. It even seems to interfere with cell migration induced by Dox. However, GE acted exactly opposite toward NIH-3T3 normal fibroblast cells and produced an anti-senescence effect. This result suggests that it may be able to prevent cell ageing and degenerative diseases in normal cells. In conclusion, the overall results showed that GE could be prepared as a co-chemotherapeutic agent and as an alternative agent for anti-ageing in healthy tissues. 

**Figure 3:**
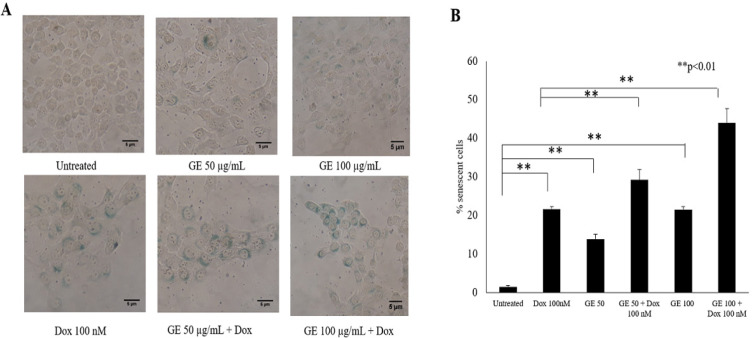
Senescence Induction Following GE Treatment in 4T1 Cells. Senescence cells were analyzed through SA-βgalactosidase staining assay. 4T1 cells (1.5 × 10^5^ cells/mL) were treated with GE 50 and 100 μg/mL alone and in combination with Dox (100 nM) for 24 h, then subjected to β-galactosidase staining. The percentages of senescent cells (β-galactosidase-positive cells) were calculated (n = 3). (A) The morphology of cells after 72 h staining under an inverted microscope with a magnification of 200x. (B) The percentage of senescent cells after treatment. Statistical significance was determined using a t-test

**Figure 4:**
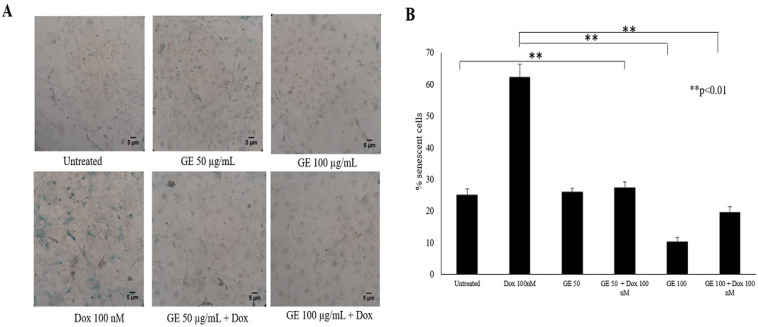
Induction of Senescence Following GE Treatment of NIH-3T3 Cells. Senescent cells were analyzed using SA-βgalactosidase staining assay. NIH-3T3 cells as the normal model (5 × 10^5^ cells/mL) were treated with GE 50 μg/mL alone and in combination with Dox (100 nM) for 24 h, then subjected to β-galactosidase staining. As a positive control, cells were treated with Dox (100 nM) for 24 h. The percentages of senescent cells (β-galactosidase-positive cells) were calculated (n = 3). (A) The morphology of cells after 72 h staining under an inverted microscope with a magnification of 200x. (B) The percentage of senescent cells after treatment

**Figure 5:**
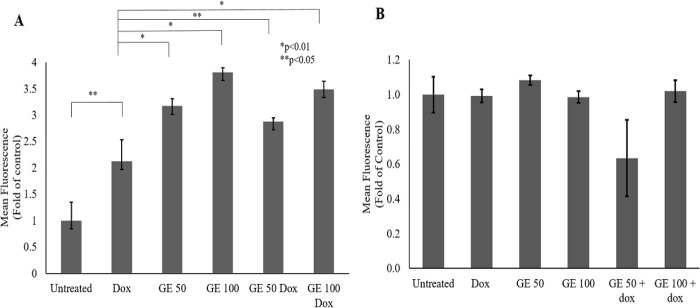
Effect of GE on Intracellular ROS Level in 4T1 and NIH-3T3 Cells. The total ROS level after a single and combination treatment of GE with Dox were observed with a DCFDA staining assay. 4T1 and NIH-3T3 cells (5 × 10^4 ^cells/mL) were treated with GE 50 and 100 μg/mL, Dox (100 nM), and in combination for 4 h, then subjected to ROS detection using flow cytometry (n = 3). (A) ROS level profile of 4T1 cells on indicated treatments (b) ROS level profile of NIH-3T3 cells on indicated treatments. The significance between treatments was determined using a t-test

**Figure 6:**
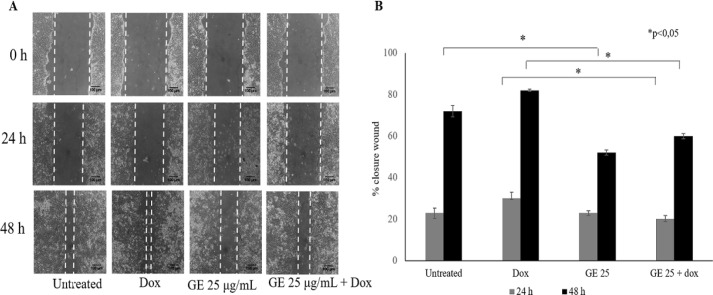
Inhibitory Effects of GE Treatment on the Cells Migration of 4T1 Cells. The cells migration effect was evaluated using a scratch wound healing assay. 4T1 cells (7.5 × 10^5^ cells/mL) were scratched then treated with GE 50 μg/mL alone and in combination with Dox (10 nM). (A) The morphology of the cells after they were scratched and treated with GE for 0, 24, and 48 h under an inverted microscope with a magnification of 100x. (B) The percentage of 4T1 cells closure after treatment. The area of closure was measured using Image J (n = 3). The percent closure was analyzed by statistical analysis (p < 0.05).

**Figure 7:**
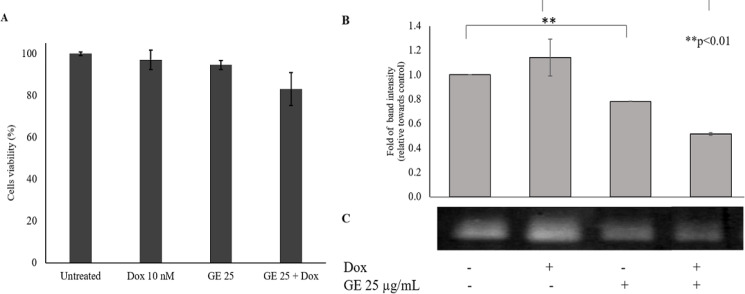
The Effect of GE Treatment on MMP-9 Expression in 4T1 Cells. MMP-9 expression was observed with a gelatin zymography assay. 4T1 cells (2 x 10^5^ cell/mL) treated with GE 25 μg/mL and in combination with Dox (10 nM) for 24 h. (A) Cell viability after treatment with GE 25 μg/mL and in combination with Dox. (B) The quantification of band intensity of MMP-9 expression. (C) Band profile of MMP-9 expression. Band intensities were quantified using ImageJ (n = 3). The percent band intensity was analyzed using the t-test procedure analysis (p < 0.05)

**Table 1:** Major Compounds of GE Analyzed by Gas Chromatography-Mass Spectrometry

Peak	Retention time (min)	Abundance (%)	Compound	Activity	Reference
1	9.676	0.56	Alpha pinene	Synergistic antitumor effect with paclitaxel	(Zhang et al., 2015)
2	11.365	0.33	Beta pinene	Synergistic antitumor effect with paclitaxel	(Zhang et al., 2015)
3	13.32	0.45	Limonene	Cytotoxic	(Oliveira et al., 2015)
4	13.427	3.18	1,8 cineole	Cytotoxic	(Sampath et al., 2017)
11	25.231	1.67	Methyl eugenol	Cytotoxic	(Yin et al., 2018)
13	25.841	7.27	Beta caryophyllene	Cytotoxic	(Legault and Pichette, 2007)
19	27.503	6.83	Germacrene	Cytotoxic potential against HL-60 cells	(da Silva et al., 2013)
23	28.149	13.95	Beta-bisabolene	Cytotoxic	(Yeo et al., 2016)
24	28.321	0.84	Eugenol	Induce apoptotic	(Al-Sharif et al., 2013)
26	28.598	17.25	Beta-sesquiphellandrene	Cytotoxic	(Tyagi et al., 2015)
29	30.155	0.37	Caryophyllene oxide	Cytotoxic	(Pan et al., 2016)

**Figure 8:**
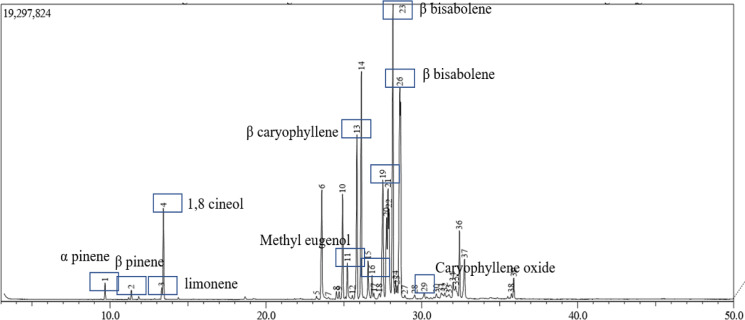
Chromatogram of GE-Containing Compounds. GE extract was analyzed using gas chromatography-mass spectrometry as described in the Methods

## Abbreviations

ACA = acetoxychavicol acetate

CYP = cytochrome

DCFDA = 2’,7’–dichlorofluorescin diacetate

Dox = doxorubicin

FBS = fetal bovine serum

GC-MS = gas chromatography-mass spectrometry

GE = galangal extract

MMP = matrix metalloproteinase

MTT = 3-(4,5-Dimethylthiazol-2-yl)-2,5-diphenyltetrazolium bromide

PBS = phosphate buffered saline

ROS = reactive oxygen species

SD = standard deviation

TNF = tumor necrosis factor
